# Intra-arterial Bevacizumab for Posterior Fossa Hemangioblastoma

**DOI:** 10.7759/cureus.32624

**Published:** 2022-12-17

**Authors:** Zachary Sokol, Ava Hoeft, David Kung, Neil Belman, Martin Oselkin

**Affiliations:** 1 Neurological Surgery, Temple University, Philadelphia, USA; 2 Neurological Surgery, St. Luke's University Health Network, Bethlehem, USA; 3 Neurological Surgery, Southern Illinois University School of Medicine, Springfield, USA; 4 Neurological Surgery, Cooperman Barnabas Medical Center, Livingston, USA; 5 Oncology, St. Luke's University Health Network, Bethlehem, USA; 6 Radiology, St. Luke's University Health Network, Bethlehem, USA

**Keywords:** endovascular, neurosurgery, von hippel landau, selective chemotherapy, posterior fossa tumor, bevacizumab, hemangioblastoma

## Abstract

Hemangioblastoma (HB) is a rare, highly vascularized, and benign central nervous system (CNS) tumor. This vascularity is due to a high degree of signaling by vascular endothelial growth factor (VEGF). Consequently, anti-VEGF agents, such as bevacizumab, have been postulated and shown in a few cases to be effective in treating these tumors when surgical therapy is not feasible. Additionally, selective intra-arterial (IA) administration of bevacizumab has shown promise in treating other cancers such as glioblastoma (GBM). Here, we present the case of a 60-year-old female with a symptomatic posterior fossa HB where embolization and surgery were not feasible due to tumor location. She underwent selective IA treatment with bevacizumab, which led to tumor stability and symptomatic improvement. Bevacizumab has been used intravenously (IV) as a treatment for HB, however, its efficacy has not been well-established. This case demonstrates the potential viability of selective bevacizumab in HB, as demonstrated by symptomatic improvement and decreased tumor size on MRI. Further research is needed to demonstrate the specific efficacy of IA bevacizumab for CNS HB when surgery or other treatment modalities are not viable options.

## Introduction

Hemangioblastoma (HB) is a rare, benign tumor of the central nervous system (CNS) that accounts for approximately 2% of all intracranial tumors. It is a highly vascular tumor. The stromal cells are the main neoplastic component and have high expression of vascular endothelial growth factor (VEGF), which accounts for their vascularity [1]. These tumors can arise sporadically, or because of von Hippel-Lindau (VHL) syndrome. Although the exact etiology remains unknown, loss of the VHL tumor suppressor gene is implicated in both the sporadic and familial forms of HB [1].

As the tumor enlarges, it may cause neurological symptoms. Surgical resection is the standard of care, and radiosurgery has been used with positive results, although its use remains controversial [1]. However, these interventions are not without risks, and not all tumors are able to be resected safely or are unresponsive to radiation. In such cases, new treatments are being explored, such as chemotherapeutic and targeted biologic agents for both HB and other CNS tumors such as glioblastoma [2-7].

Chemotherapeutic agents, such as thalidomide, have been explored as options for reducing angiogenesis in HB in familial and pediatric cases. Biologic agents, such as the VEGF inhibitor bevacizumab, have shown promise in the treatment of HB, although evidence is sparse and its use has been through an intravenous (IV) route [5,7]. Intra arterial (IA) bevacizumab has shown promise for other primary brain malignancies such as glioblastoma (GBM) [2,3,6].

The patient described here experienced clinical benefit, although with some side effects. She demonstrated radiological stabilization of symptomatic HB with bevacizumab.

## Case presentation

**Figure 1:**
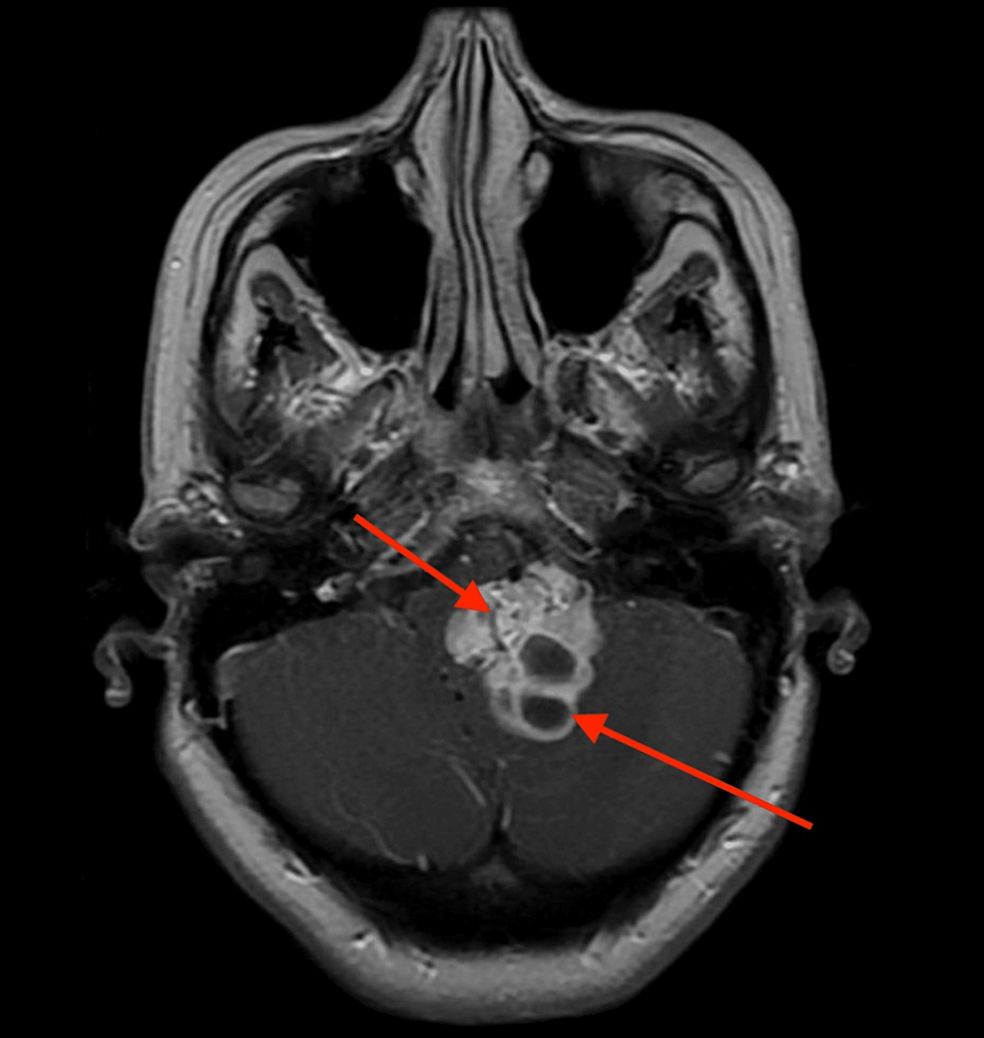
Post-contrast T1-weighted MRI reveals an irregular, mixed solid and cystic avidly enhancing mass centered in the foramen of Luschka that measured approximately 3.5 x 3.0 x 4.2 cm​

Our patient was a 60-year-old female with a history of falls and gait decline in 2016. She underwent an MRI, which revealed a large posterior fossa mass with solid and cystic components (Figure 1). She was transferred to a large urban academic center. Embolization of the mass was not performed due to the risk of infarction to the brainstem. She did undergo surgery but tumor resection was aborted due to the intimate association of the medulla and the hypoglossal nerve. A biopsy and a craniectomy with duraplasty were performed instead. Pathology of the mass was consistent with a WHO grade I HB. Genetic testing was negative for familial VHL. While in rehabilitation, she developed progressive hydrocephalus requiring ventriculoperitoneal shunt placement. Given the inoperable nature of the mass, external beam radiation therapy was performed for a total of 54 Grays of radiation over 30 fractions completed in 2017. By 2018, the patient was still suffering from nystagmus, ataxia, and left-sided weakness. Throughout this time, surveillance imaging revealed expected post-treatment changes, however, the tumor continued to enlarge, predominantly the cystic portion. After exhausting conventional treatments, hospice was considered. Following a multidisciplinary discussion with Oncology, Neurosurgery, and Palliative Care, it was decided to offer her intra-arterial (IA) bevacizumab for salvage therapy followed by IV bevacizumab for maintenance.

The patient underwent cerebral angiography via the femoral approach. The left vertebral artery was selected with an SL-10 microcatheter and positioned at the left V3/V4 junction. Angiography at this level revealed preferential flow into the tumor with significant contrast opacification. Ten mg/kg of bevacizumab was slowly infused via the microcatheter over the course of 15 minutes, for a total of 700 mg of bevacizumab. The patient subsequently received biweekly IA bevacizumab, for a total of six treatments, via alternating femoral approaches. After each treatment, the patient experienced varying degrees of worsening nystagmus, diplopia, headaches, lower back pain, nausea, and vomiting, which typically resolved within 24 hours.

**Figure 2:**
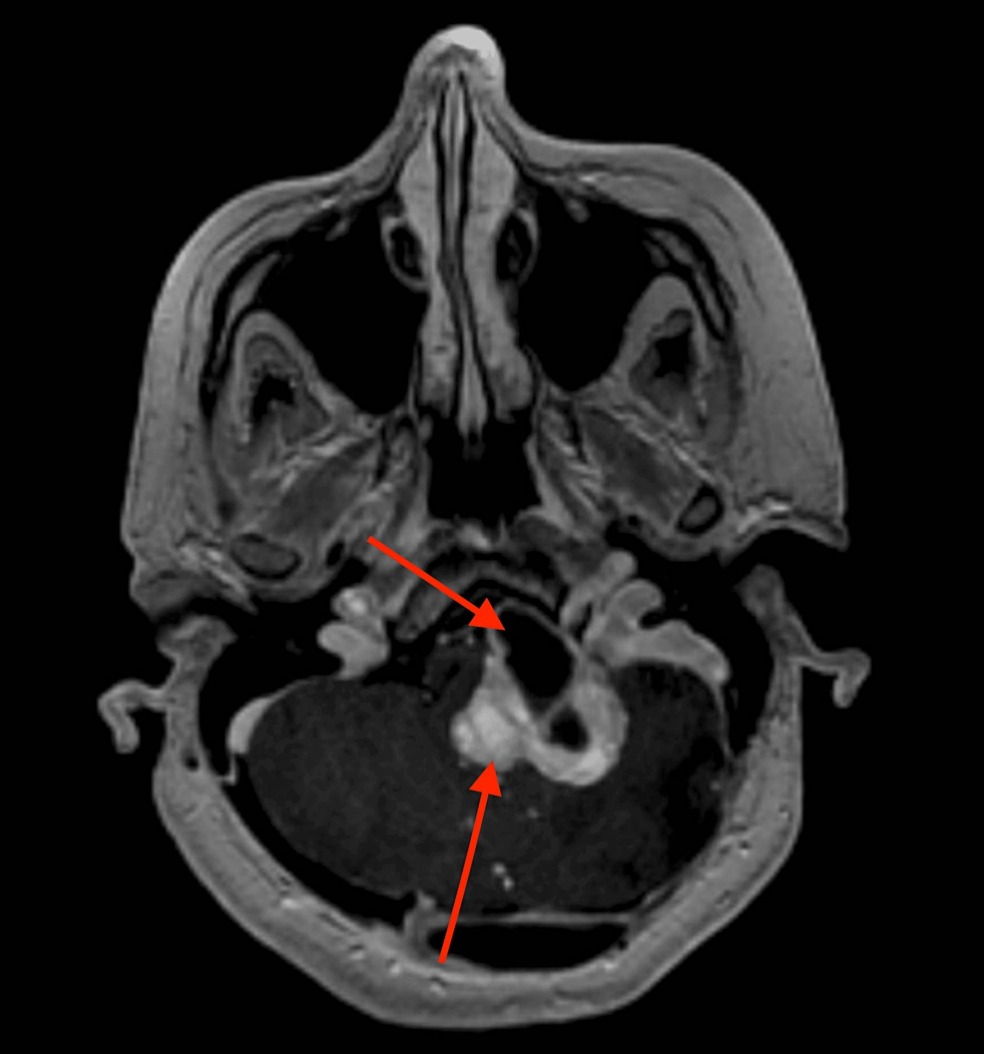
Comparative axial image obtained after intra-arterial bevacizumab therapy now demonstrates the tumor measuring 3.59 x 3.19 cm with a decreased thickness of the enhancing tumor surrounding the cystic portion

**Figure 3:**
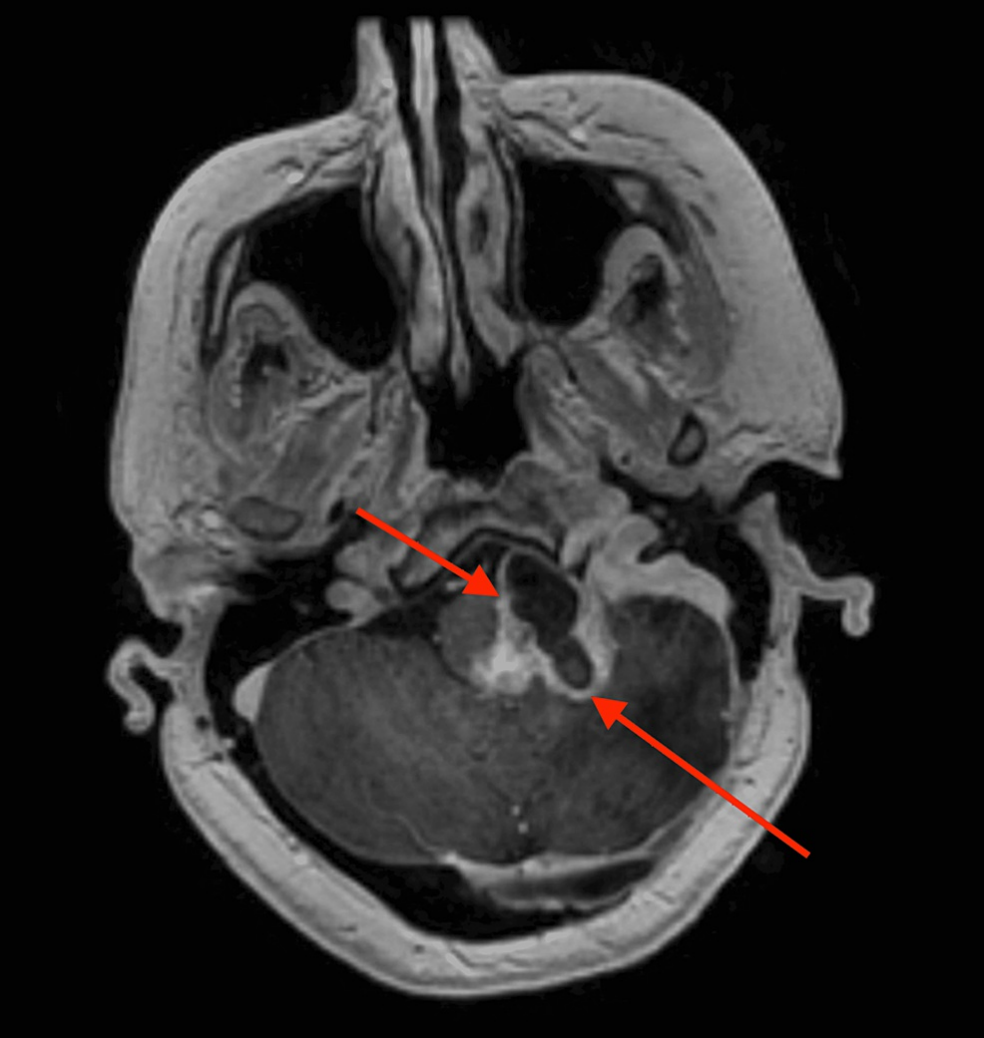
Comparative axial image obtained after intra-arterial bevacizumab therapy now demonstrates the tumor measuring 3.59 x 3.19 cm with a decreased thickness of the enhancing tumor surrounding the cystic portion

**Figure 4:**
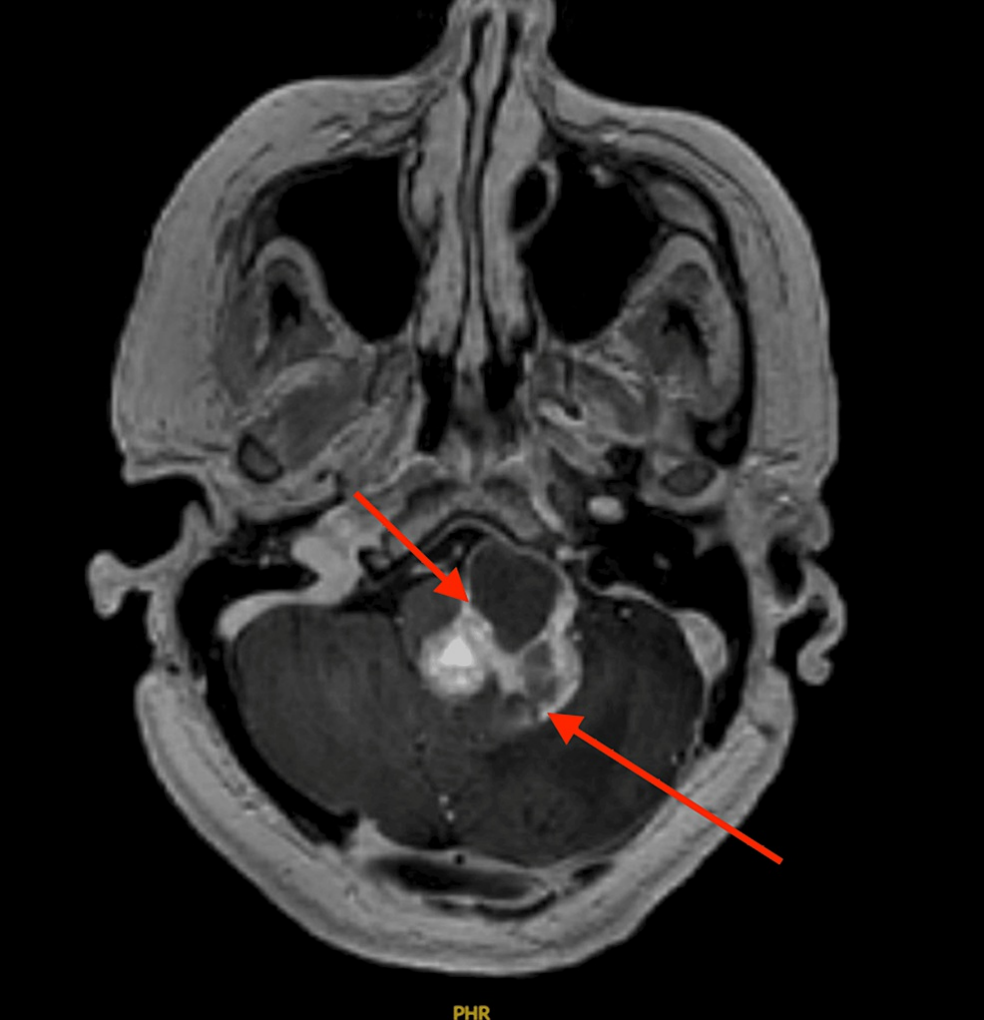
Baseline MRI prior to the new course of intra-arterial bevacizumab therapy reveals a similar appearance of the tumor now measuring 4.08 x 3.51 cm

**Figure 5:**
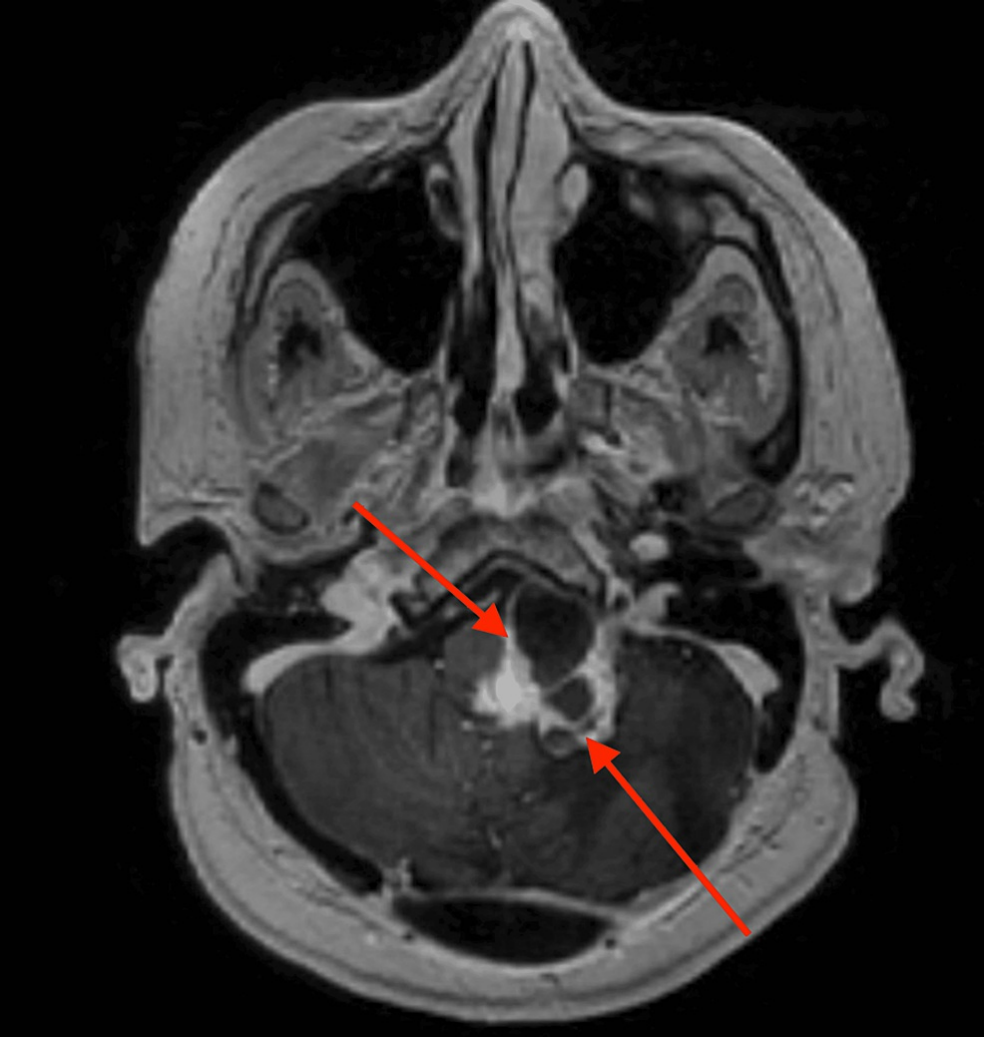
Comparative axial image obtained after intra-arterial bevacizumab therapy now demonstrates the tumor measuring 3.44 x 3.16 cm

After the completion of IA therapy, a surveillance MRI was performed in an outpatient setting. At her three-month MRI, there was an approximately 20% tumor volume reduction when compared to her baseline imaging (Figure 2, Figure 3) as well as decreased contrast avidity. The patient underwent venous port insertion for maintenance IV bevacizumab therapy eight weeks after her last arterial infusion, with the first infusion two weeks after port insertion. Shortly thereafter, the patient suffered a canine attack on her chest and experienced wound dehiscence that required port removal. As a result, IV therapy was halted. Surveillance imaging resumed and brain MRI again showed a recurrence of disease progression seven months after her last IV dose. She became more symptomatic with worsening fatigue and headaches. We again offered her IA bevacizumab followed by IV maintenance. Due to the COVID-19 pandemic and concern for immunosuppression, the new regimen of IA bevacizumab was delayed until 2021 following COVID-19 vaccination. To minimize her exposure to the hospital environment, an IA infusion every three weeks was planned. The patient received 15 mg/kg every three weeks for a total of four rounds via alternating radial approaches. Follow-up MRI at three months revealed the tumor had again diminished in size, but there remained a significant mass effect on the medulla (Figure 4, Figure 5). The patient was subsequently tapered to IV bevacizumab infusion.

## Discussion

HB is a highly vascularized tumor that can arise in the retina, cerebral hemispheres, brain stem, and spinal cord. The pathogenesis is not well-understood, but mutations in the VHL gene, located on chromosome 3p25-26, have been implicated. The protein coded by this gene (pVHL) is crucial for the degradation of the hypoxia-inducible factor (HIF) transcription complex. One of the factors that HIF upregulates is VEGF. Loss of VHL thus leads to uncontrolled HIF signaling, which increases VEGF production [1]. Since VEGF and its receptors are implicated in HB tumorigenesis, we hypothesized that the use of IA bevacizumab would help control the progression of this tumor.

**Figure 6:**
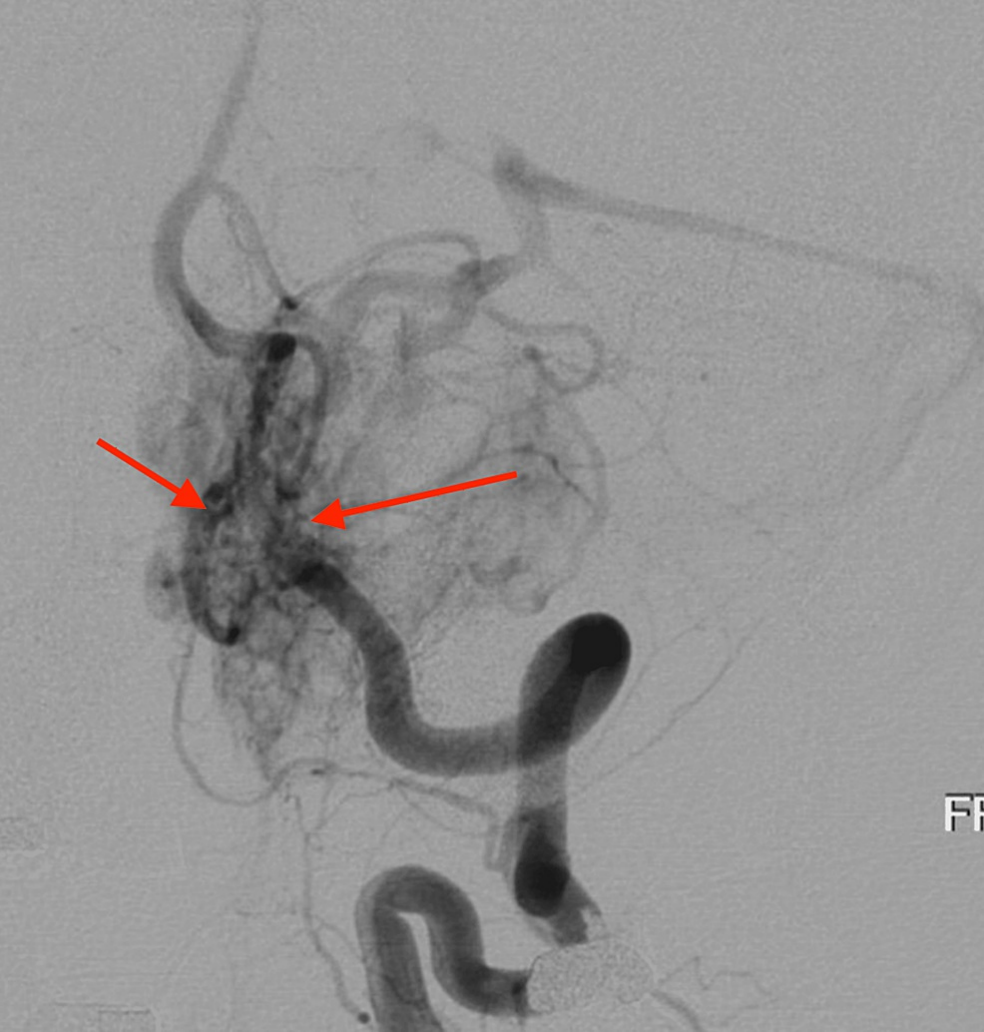
Cerebral angiography via a left vertebral artery injection The frontal view reveals marked vascularity of the mass with some early shunting.

Our treatment protocol was based on prior reports of IA bevacizumab for GBM from Riina et al. and D’amico et al., with some exceptions [3,6]. First, we did not employ blood brain barrier (BBB) disruption with mannitol. BBB disruption was not performed because HB is a neoplasm of blood vessels, as opposed to the brain parenchyma, and thus disruption of the BBB was not necessary. Furthermore, we believed that evidence of avid enhancement on MRI and angiographic vascularity (Figure 6) was a sign that the barrier was already disrupted locally and there would be high penetrance of bevacizumab into the neoplastic stroma.

We did not utilize a superselective microcatheter position. There was no dominant arterial feeder to the tumor, rather, there were numerous short and fine branches. We did not utilize a balloon occlusion microcatheter as previously described by Riina et al. [6]. Similar to our reasoning for no BBB disruption, we felt that high-contrast uptake on MRI and vascularity on angiography eliminated the need for a balloon occlusion catheter inflated in the basilar artery to shunt blood flow and therefore bevacizumab proximally into the tumor. This also prevented the risk of basilar perforator stroke from prolonged occlusion.

This therapy was effective in shrinking the size of the tumor and preventing further growth. However, this was in a single patient and the patient did experience some side effects. MRI improvement related to decreasing enhancement was similar to what was demonstrated on MRI with GBM IA bevacizumab infusion with BBB disruption, confirming that bevacizumab would have high penetrance into the neoplastic stroma on account of its highly vascularized nature. Our patient experienced wound dehiscence, which is a known complication of bevacizumab treatment, and thus caution must be exercised in patients who receive this therapy [8].

## Conclusions

The use of IA bevacizumab in treating otherwise unresponsive HB remains relatively unexplored. In this patient, the use of IA bevacizumab was successful in controlling the growth of the tumor, with minimal side effects. We demonstrate its effectiveness without BBB disruption in a patient indicating the possibility of further use of this technique. With described cases of IV bevacizumab treatment, and the IA treatment techniques in use for GBM, we propose that further studies be conducted on the efficacy of IA bevacizumab to demonstrate its potential for treatment in HB unresponsive or ineligible for conventional resection and stereotactic radiosurgery (SRS), as well as the potential for utilization of other VEGF-targeted therapies.
